# The hidden base of the iceberg: gut peptidoglycome dynamics is foundational to its influence on the host

**DOI:** 10.1080/19490976.2024.2395099

**Published:** 2024-09-06

**Authors:** Richard Wheeler, Ivo Gomperts Boneca

**Affiliations:** a Institut Pasteur, Université Paris Cité, Paris, France; b Hauts-de-Seine, Arthritis Research and Development, Neuilly-sur-Seine, France

**Keywords:** Microbiota, peptidoglycan, prophage, host signaling, chronic inflammation, Gut, peptidoglycan processing, PG trafficking

## Abstract

The intestinal microbiota of humans includes a highly diverse range of bacterial species. All these bacteria possess a cell wall, composed primarily of the macromolecule peptidoglycan. As such, the gut also harbors an abundant and varied peptidoglycome. A remarkable range of host physiological pathways are regulated by peptidoglycan fragments that originate from the gut microbiota and enter the host system. Interactions between the host system and peptidoglycan can influence physiological development and homeostasis, promote health, or contribute to inflammatory disease. Underlying these effects is the interplay between microbiota composition and enzymatic processes that shape the intestinal peptidoglycome, dictating the types of peptidoglycan generated, that subsequently cross the gut barrier. In this review, we highlight and discuss the hidden and emerging functional aspects of the microbiome, i.e. the hidden base of the iceberg, that modulate the composition of gut peptidoglycan, and how these fundamental processes are drivers of physiological outcomes for the host.

## Introduction

In 1982, James Kruger, John Pappenheimer and Manfred Karnovsky identified a sleep-promoting small molecule in the cerebral spinal fluid and urine of humans and animals^1^. This sleeping factor turned out to be a soluble muramyl peptide originating from the peptidoglycan network that forms the major part of the cell wall of bacteria. Kruger, Pappenheimer and Karnovsky suggested that muramyl peptides present in host body fluids could originate from the bacteria that inhabit the intestinal tract. Over forty years later, the peptidoglycan generated in our gut by the bacterial flora, is finally beginning to be recognized as a major player in the communication axis between the intestinal microbiota and host. The commensal microbiota of the intestinal tract comprises in the order of 10^13^ individual bacteria, encompassing many hundreds of bacterial species^2^ that collectively produce different varieties of peptidoglycan, and thus, the human intestinal tract harbors a potentially enormous diversity of peptidoglycan molecules. We can think of the peptidoglycan collectively produced by all gut-resident bacteria combined as our “intestinal peptidoglycome”. Peptidoglycan belongs to the group of Microbe Associated Molecular Patterns (MAMPs), which includes several components of the bacterial cell envelope that are shed naturally into the surrounding environment, or due to attack, death and lysis of bacteria by host immune effectors or competition with other members of the microbiota. As with all MAMPs, peptidoglycan moieties are sensed directly by the host innate immune system through dedicated pattern recognition receptors. The best studied and characterized sensors of peptidoglycan are the cytosolic receptors NOD1 and NOD2, for which the molecular requirements for recognition of specific muropeptides (soluble peptidoglycan fragments) are well defined, the minimal structural motifs being D-isoglutamine-*meso*diaminopimelic acid (iE-DAP) for NOD1 and *N*-acetylmuramic acid-L-Alanine-D-isoglutamine (MDP) for NOD2.^3–9^ However, the relative simplicity of this sensor system belies the diversity and complexity of host responses to peptidoglycan.

**Figure 1.**
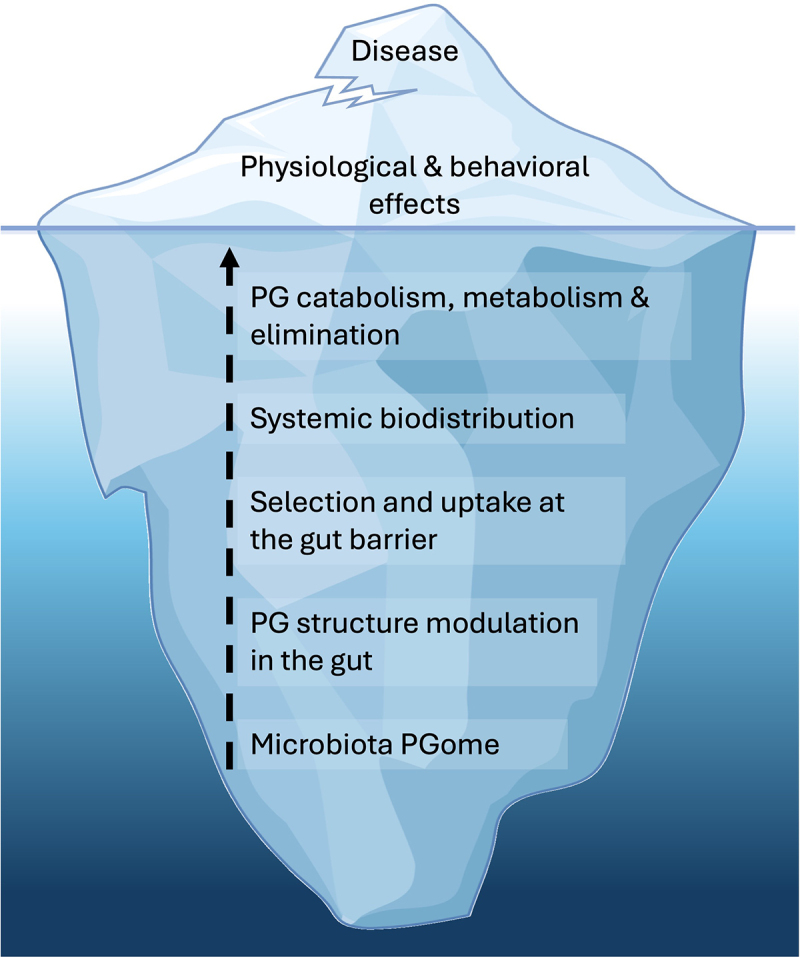
The “iceberg” of gut peptidoglycome dynamics in the host.

**Figure 2.**
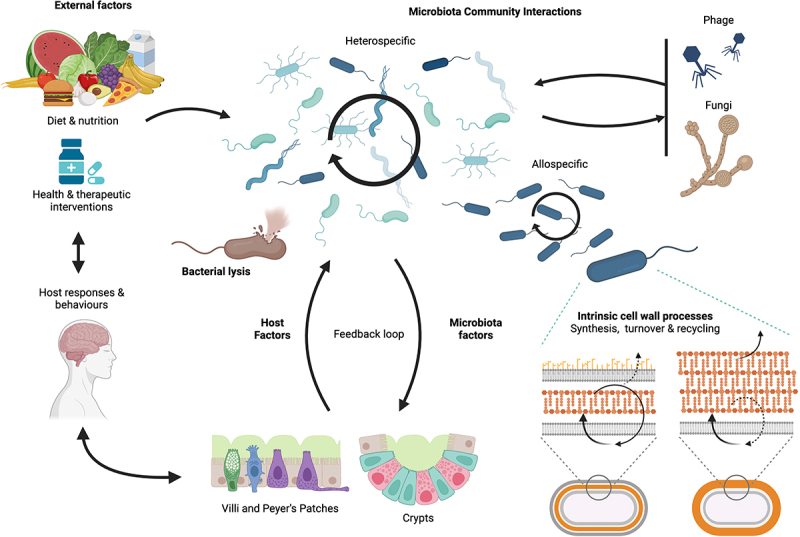
Interaction and feedback between major parameters of the gut ecosystem that shape the intestinal peptidoglycome.

In recent years, there has been a rise in studies detailing the effects that microbiota peptidoglycan exerts over the host. These effects can be considered the tip of a gut peptidoglycome iceberg – observable due to the impact they have on the host, such as changes to host behaviors, physiological development and function, and effect on immune activity (Figure 1). Most visible at the very tip of the peptidoglycome iceberg, is when the host suffers pathological consequences from breakdown of homeostasis between microbiota peptidoglycan and its sensing by the host, leading to disease. Effects of peptidoglycan on the host have typically been observed and probed using a few commercially available NOD ligands with well-defined spectra of activity, administered experimentally as surrogates of the gut peptidoglycan. Our understanding of the natural cycle of the gut microbiota peptidoglycan and its interactions with the host are lacking. Thus, if the tip of the iceberg represents the observable effects of gut peptidoglycan on the host, then hidden from sight beneath the surface lies the enormous base of the peptidoglycome iceberg (Figure 1). At the very foundation of the iceberg is the chemical nature of peptidoglycan generated by the gut bacteria themselves (Figures 1 and 2). This is followed by the dynamics of peptidoglycan liberation within the gut lumen, and the structural modulation of peptidoglycan fragments by neighboring commensal organisms and by host enzymes that determine the peptidoglycan structural moieties that reach the interface with the gut barrier (Figures 1 and 2). Here, there is the potential for selection of specific structural moieties via different mechanisms of uptake at the epithelial interface, and trafficking into channels of systemic biodistribution (Figures 1 and 2). Within the host system, very little is understood about the catabolism or metabolism of different peptidoglycan chemical moieties, and how these might be sensed, modified and eventually eliminated from the host. Finally, from the moment peptidoglycan fragments come into contact with the gut epithelial cells, there is an opportunity for host sensing of classical and non-canonical peptidoglycan fragments by immune and nonimmune cells in different organs and tissue compartments, where peptidoglycan effects on the host are initiated (Figures 1 and 2). Thus, within the hidden base of the peptidoglycome iceberg, there is a rich and complex world remaining to be explored and studied, to understand the full extent to which microbiota peptidoglycan can influence the host.

In this review we aim to highlight recent studies that have begun to scratch beneath the surface of the peptidoglycome iceberg, focusing on the nature of the peptidoglycan molecules naturally generated by the microbiota within the gut niche, and the different mechanisms shaping their influence over the host.

### The base of the iceberg: the composition and diversity of gut microbiota peptidoglycan

**Figure 3.**
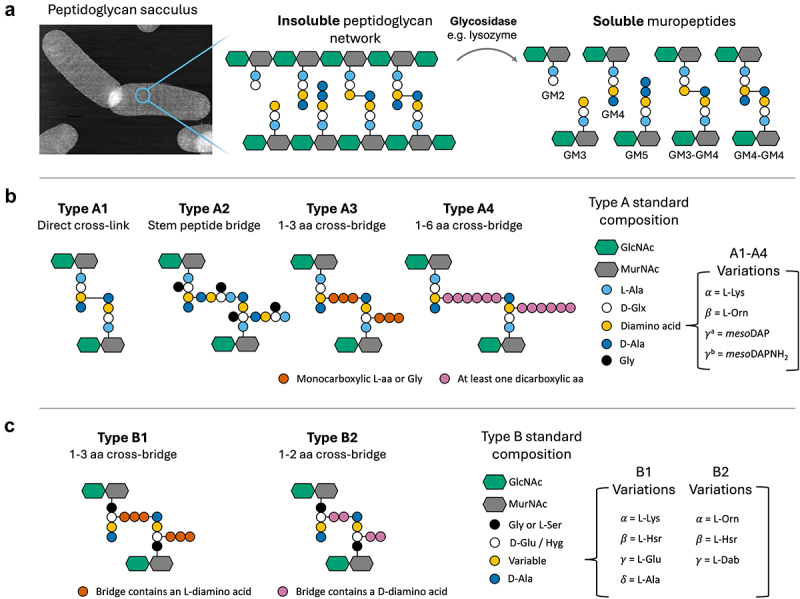
Summary diagram of the peptidoglycan network and the peptidoglycan types and variants according to the scheme of Schleifer and Kandler.^10^ A; simple schematic of the peptidoglycan polymer and its soluble fractions, “muropeptides”, generated by glycosidase activity. Image shows a typical diderm peptidoglycan sacculus (atomic force microscopy height profile image of purified *Helicobacter pylori* peptidoglycan). B; schematic of type A peptidoglycan, represented based on a GM4 dimer. The most typical variants are indicated. C; schematic of type B peptidoglycan, represented based on a GM4 dimer. The most typical variants are indicated. Ala, alanine; dab, diaminobutyric acid; glu, isoglutamate; glx, isoglutamate or isoglutamine; gly, glycine; Hsr, homoserine; Hyg, threo-3-hydroxyglutamate; lys, lysine; *meso*DAP, *meso*-diaminopimelic acid; *meso*DAPNH_2,_ amidated *meso*-diaminopimelic acid; orn, ornithine.

The bacterial cell is surrounded by a macromolecular peptidoglycan network, referred to as a sacculus. It has two distinct components – chains of glycan and peptide cross-links (Figure 3(a)). The glycan comprises a repeating disaccharide of *N*-acetyl-D-glucosamine (GlcNAc) and *N*-acetylmuramic acid (MurNAc) linked by beta-1,4 glycosidic bonds. Within the sacculus, glycan chains can be very short to several hundred nanometers in length.^11,12^ The glycan chains are cross-linked by short peptides, *N*-linked to the lactyl moiety of MurNAc, and comprising L- and D-amino acid enantiomers. By far the most common peptide sequence is L-Alanine, D-glutamine/glutamate, a diamino acid (most commonly *meso*-diaminopimelate or L-lysine), and D-alanyl-D-alanine at the C-terminus. The network-like structure of peptidoglycan is bestowed by cross-linking between the peptide-stems attached to adjacent glycan strands. The presence of the diamino acid is crucial to peptide cross-linking, which most commonly occurs via a diamino acid at position three of the peptide stem, and the fourth amino acid, D-alanine, present at the C-terminus of the adjacent peptide stem (following cleavage of the terminal D-Alanine at position 5), although 3–1, 3–3 and 2–4 cross-linking can also occur.^10,13,14^ Indirect cross-linking of adjacent peptide stems is common in monoderm bacteria, mediated by peptide bridges up to 6 amino acids in length^10^ (Figure 3(b)).

Although the general composition of peptidoglycan is relatively simple, a great diversity of structures is found throughout the bacterial kingdom. In the 1970s, Karl Schleifer and Otto Kandler compiled a comprehensive cataloging of peptidoglycans, and proposed a classification system placing peptidoglycan into two major groups (A and B), six subgroups (A1-A4, B1-B2) and a multitude of subtypes within most of these group^10^ (Figure 3(b,c)). Additional peptidoglycan types have been identified subsequently.^15,16^ Based on studies of the typical diversity of the human bacterial flora, and decades of peptidoglycan composition analysis of culturable bacteria, we can infer that peptidoglycan types belonging to subgroups A1, A3, A4, and B2 are represented in the human gut peptidoglycome (Table 1). Over ninety percent of human gut bacteria belong to two phyla, the Bacteroidota (formerly Bacteroidetes) and Bacillota (formerly Firmicutes).^56–59^ These phyla represent two extremes in terms of their peptidoglycan diversity. Peptidoglycan composition is highly diverse among Bacillota, and the gut-resident members of this phylum are expected to include A1γ, A3α, A4α, A4β and B2α peptidoglycan types (Table 1). Experimentally, peptidoglycan of types A1γ, A3α, A4α and A4β have been reported by LC-MS analysis of cultured species of common human gut resident bacteria, or from peptidoglycan sacculi extracted from mouse fecal microbiota.^60,61^ The A3 and A4 peptidoglycan types are particularly diverse due to the presence of peptide cross-bridges from one to six amino acids in length, whose amino acid sequence can vary even between closely related species.^10^ In contrast, peptidoglycan diversity is comparatively low among members of the Bacteroidota, since their cells walls are expected to almost exclusively comprise the directly cross-linked A1γ peptidoglycan, which predominates among the diderm bacteria (Table 1). Exceptions include several species of spirochete, reported to comprise Type A peptidoglycan with ornithine (A3β) instead of *meso*DAP as the dibasic amino acid, and glycine crossbridge.^62–64^ However, spirochetes are not abundant in the gut and may therefore represent, at most, a very minor portion of the gut microbiota peptidoglycome.

**Table 1.** Expected PG diversity of a “baseline” human gut microbiota, based on their characterized peptidoglycan types. The table presents peptidoglycan types, using the classification system of Schleifer and Kandler, identified from *in vitro* peptidoglycan characterization studies of bacteria belonging to genera present in the human microbiota.^10^ Gut microbiota genera are from the baseline healthy human gut microbiota described by King *et al*.^66^ Where the precise PG type has not been defined, the diamino acid (DAA) is indicated where possible.

Phylum	Class	Order	Family	Genus	Type	Reported Variants	Refs
Actinobacteria/ Actinomycetota	Actinobacteria/ Actinomycetia	Actinomycetales	Corynebacteriaceae	Corynebacterium	A, B	(A)1γ 4α (B)2β	^10,17^
		Bifidobacteriales	Bifidobacteriaceae	Bifidobacterium	A	3α 3β 4γ	^10,18,19^
				Gardnerella		DAA: Lysine	^20^
		Propionibacteriales	Propionibacteriaceae	Cutibacterium	A	1γ (_LL_-DAP) 3γ	^21^
		Streptomycetales	Streptomycetaceae	Streptomyces	A	3γ (_LL_-DAP)	^22^
	Coriobacteriia	Coriobacteriales	Coriobacteriaceae	Atopobium	A	4α	^23^
		Eggerthellales	Eggerthellaceae	Adlercreutzia	-	-	-
				Gordonibacter	-	-	-
Firmicutes/ Bacillota	Clostridia	Clostridiales	Clostridiaceae	Clostridium	A	1γ DAA:Lys	^24,25^
			Eubacteriaceae	Eubacterium	A	1γ, 4α, 4β	^26–29^
					B	2α	
			Lachnospiraceae	Roseburia	-	-	-
				Blautia	-	DAA: *meso*DAP	^30^
				Coprococcus	-	-	-
				Lachnoclostridium	-	-	-
			Ruminococcaceae	Ruminococcus	-	-	-
				Ruminiclostridium	-	-	-
				Ethanoligenens	-	-	-
				Mageeibacillus	-	-	-
			Peptostreptococcaceae	Clostridioides	-	-	-
				Paeniclostridium	-	-	-
		Eubacteriales	Oscillospiraceae	Faecalibacterium	-	DAA: *meso*DAP	^31^
				Oscillibacter	-	-	-
	Negativicutes	Veillonellales	Veillonaceae	Dialister	-	-	-
				Veillonella	A	1γ	^32^
		Selenmonadales	Selenomonadaceae	Megamonas	-	-	-
		Acidaminococcales	Acidaminococcaceae	Acidaminococcus	-	-	-
	Tissierellia	Tissierellales	Peptoiphilaceae	Anaerococcus	A	4α	^15^
				Parvimonas	-	-	-
	Bacilli	Bacillales	Bacillaceae	Bacillus	A	1γ	^33^
			Paenibacillaceae	Paenibacillus	A	1γ	^34^
			Staphylococcaceae	Staphylococcus	A	3α	^35–37^
		Lactobacillales	Lactobacillaceae	Lactobacillus	A	1γ, 4α, 4β	^38–40^
			Streptococcaceae	Streptococcus	A	3α, 4α	^41,42^
				Lactococcus	A	4α	^43,44^
			Enterococcaceae	Enterococcus	A	3α 4α	^45^
			Leuconostocaceae	Leuconostoc	-	-	-
	Sphingobacteriia	Sphinobacteriales	Sphingobacteriaceae	Sphingobacterium	-	-	-
Bacteroidetes/ Bacteroidota	Bacteroidia	Bacteroidales	Bacteroidaceae	Bacteroides	A	1γ, 4γ	^46^
			Barnesiellaceae	Barnesiella	-	-	-
			Porphyromonadaceae	Fermentimonas	-	-	-
			Odoribacteraceae	Odoribacter	-	-	-
			Tannerellaceae	Tannerella	A	1γ	^47^
				Parabacteroides	-	-	-
			Porphyromonadaceae	Porphyromonas	-	-	-
			Rikenellaceae	Alistipes	-	-	-
			Prevotellaceae	Prevotella	-	DAA: *meso*DAP	^46^
	Flavobacteriia	Flavobacteriales	Flavobaceriaceae	Ornithobacterium	-	-	-
	Sphingobacteriia	Sphingobacteriales	Sphingobacteriaceae	Sphingobacterium	-	-	-
Pseudomonadota Proteobacteria/	Betaproteobacteria	Burkholderiales	Comamonadaceae	Acidovorax	-	-	-
	Gammaproteobacteria	Enterobacterales	Enterobacteriaceae	Escherichia	A	1γ	^32,48^
				Shigella	A	1γ	^49^
				Citrobacter	A	1γ	^50^
				Klebsiella	A	1γ	^51^
				Raoultella	-	-	-
		Pasteurellales	Pasteurellaceae	Haemophilus	A	1γ	^52^
Myxococcota	Deltaproteobacteria	Desulfovibionales	Desulfovibionaceae	Desulfovibrio	-	-	-
				Bilophila	-	-	-
Campylobacterota	Epsilonproteobacteria	Campylobacterales	Helicobacteraceae	Helicobacter	A	1γ	^53^
			Campylobacteraceae	Campylobacter	A	1γ	^54^
Fusobacteriota	Fusobacteriia	Fusobacterialies	Fusobacteriaceae	Fusobacterium	A	1γ, 4γ (Lan)	^46^
Verrucomicrobiota	Verrucomicrobiae	Verrucomicrobales	Akkermansiaceae	Akkermansia	A	1γ	^55^
Spirochaetota	Spirochaetia	Spirochaetales	Spirochaetaceae	Treponema	A	3β	^67^
Planctomycetota	Planctomycetia	Planctomycetales	Planctomycetaceae	Rubinsphaera	-	-	-

One could imagine that the intestinal lumen is dominated by A1γ peptidoglycan of the Bacteroidota, and diderm bacteria in general, due to the collective generation of A1γ peptidoglycan fragments by almost all these bacteria. However, members of the Bacteroidetes and most other diderm bacteria have a very thin peptidoglycan sacculus, only one or a few layers thick, whereas monoderm bacteria, including the vast majority of the Bacillota, have a much thicker, multilayered peptidoglycan. Therefore, peptidoglycan of monoderm bacteria represents a greater mass per cell than peptidoglycan of diderm bacteria of the same size and shape (reviewed in,^13^ see also^65^). In support of this, LC-MS analysis of purified peptidoglycan sacculi from mouse gut microbiota suggests that major A1γ and A4α (with glutamate or glutamine peptide bridges) muropeptides, are similar in their relative abundance.^61^ Another point of note is that in monoderm bacteria with A1γ peptidoglycan, the *meso*DAP moiety is typically amidated, which abrogates its recognition by NOD1.^5^ Therefore, the potential contribution of biologically active peptidoglycan moieties likely differs between diderm and monoderm bacteria with A1γ peptidoglycan.

### Liberation of peptidoglycan muropeptides in the gut lumen

**Figure 4.**
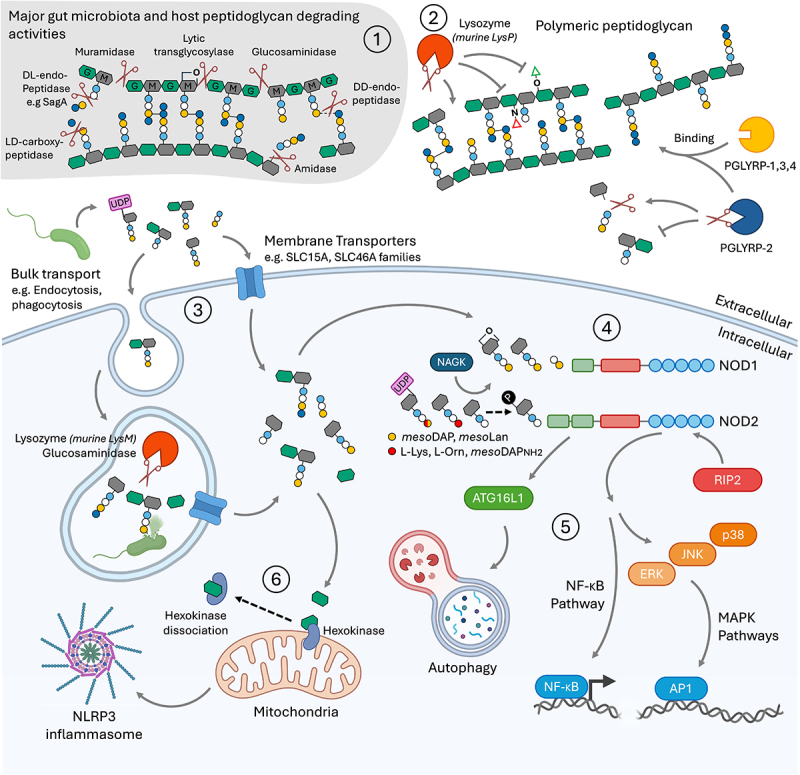
The specificity of microbiota and host enzymes and receptors for peptidoglycan. 1) the major peptidoglycan-degrading enzymatic activities of the microbiota or host discussed in this review. 2) lysozyme and PGLYRP-2 are the major host secreted enzymes capable of binding and cleaving polymeric peptidoglycan. PGLYRP-1, −3 and − 4 bind polymeric peptidoglycan but have no hydrolase activity. Green triangle, *O*-acetylation; red triangle, De-*N*-acetylation. 3) muropeptides released from bacteria must reach the eukaryotic cell cytosol to be detected. SCL15A-family and SLC46A-family membrane transporters are implicated in this function. Bulk transport of peptidoglycan or bacteria may occur via endocytosis pathways, but the muropeptides must be transported across the endosomal membrane to be sensed by PRRs. 4) the major host peptidoglycan receptors, NOD1 and NOD2, sense distinct muropeptide fractions. The minimal and classical ligands are indicated. NOD1 also senses 1,6-anhydro MurNAc-containing muropeptides whilst the presence of UDP expands the range of muropeptides recognized by NOD2. Phosphorylation of MurNAc by NAGK augments recognition by NOD2. 5) muropeptide binding triggers NOD1/2 oligomerization and recruitment of RIP2, initiating NF-κB or MAPK signaling pathways resulting in cellular response outputs. Alternatively, NOD1/2 can recruit ATG16L1 to initiate autophagy. 6) *N*-acetyl-D-glucosamine can be bound by hexokinase leading to its dissociation from the mitochondrial membrane, which triggers a pathway leading to NLRP3 inflammasome activation. Amidase, *N*-acetylmuramyl-L-alanine amidase; glucosaminidase, *N*-acetyl-β-D-glucosaminidase; Lys, lysine; *meso*DAP, *meso*-diaminopimelic acid; *meso*DAPNH_2,_ amidated *meso*-diaminopimelic acid; *meso*Lan, *meso-*lanthionine; orn, ornithine.

The insoluble peptidoglycan sacculus within the bacterial cell wall represents the majority of peptidoglycan biomass in the gut, with peptidoglycan components estimated to represent from 1.6% (*Escherichia coli*) to 14% (*Streptococcus salivarius subsp. thermophilus*) of the bacterium dry weight.^68^ Generally speaking, the host system should not be directly exposed to the insoluble peptidoglycan sacculi of gut bacteria, as the innate barrier defenses in the lumen such as the mucus, antimicrobial peptides, mucosal antibodies and lysozyme, restrict contact between the bacterial flora and the intestinal epithelial cells.^69^ Therefore, when considering the intestinal peptidoglycome, it can be useful to think of it as having two distinct parts: the “insoluble peptidoglycome”, comprising the cell wall sacculi that surround each bacterium, and the “soluble peptidoglycome”, comprising small soluble “muropeptide” peptidoglycan fragments that are naturally shed from the insoluble peptidoglycan sacculi of bacteria as a result of enzymatic cleavage as they grow and divide, or due to cell lysis. Soluble muropeptides are the classical signaling effector units recognized by host pattern recognition receptors NOD1 and NOD2^3–5,70^ (Figure 4). In this section we discuss the factors influencing how soluble peptidoglycome muropeptides are liberated by microbiota or host enzymes active in the gut.

#### Liberation of soluble muropeptides by the microbiota: synthesis, turnover and recycling of peptidoglycan

Soluble muropeptides are released from the peptidoglycan sacculus, through the action of endogenous peptidoglycan hydrolases and lyases that cleave the cell wall (Figure 4) during bacterial growth and division, in a process called peptidoglycan turnover. In diderm bacteria, the major soluble muropeptide products of peptidoglycan turnover are generated through the activity of lytic transglycosylase enzymes. These enzymes catalyze a non-hydrolytic lyase reaction, cutting the beta-1,4 glycosidic bond between MurNAc (C1) and GlcNAc (C4), with concomitant cyclization of MurNAc between C1 and C6, forming 1,6-anhydro MurNAc.^71^ Lytic transglycosylase activity is comparatively minor among the monoderm bacteria.^72^ The major glycan cleaving activities associated with monoderm peptidoglycan turnover are muramidases, which cut the beta-1,4 glycosidic bond between MurNAc (C1) and GlcNAc (C4) without modification of MurNAc. *N*-acetyl-β-D-glucosaminidases (glucosaminidases) complement the muramidases by cutting the beta-1,4 glycosidic bond specifically between GlcNAc (C1) and MurNAc (C4). Therefore, it would be expected that the presence of 1,6-anhydro-muropeptides liberated into the gut should be a hallmark of muropeptides released by diderm gut commensal bacteria. However, a recent comprehensive study of the peptidoglycan composition of monoderm bacteria from the genus *Bifidobacterium* showed that members display a high proportion of 1,6-anhydro muropeptides, suggesting that there may be exceptions among important members of the gut microbiota.^73^

1,6-anhydromuropeptides are the specific targets of efficient peptidoglycan recycling in diderm bacteria, which has mainly been studied in the Pseudomonadota (Proteobacteria), particularly in members the *Enterobacteriaceae*. Transport of 1,6-anhydromuropeptide turnover products from the periplasm to the cytosol is performed by the permease AmpG, which is widespread among the betaproteobacteria and gammaproteobacteria, and found more sporadically among the alpha-, delta- and epsilonproteobacteria.^74^ Alphaproteobacteria appear to transport peptidoglycan turnover products mainly via the YejBEF-YepA ABC transporter.^74,75^ Factors aiding efficient peptidoglycan recycling in diderms include the fact that the peptidoglycan is at most a few layers thick, and that turnover occurs in the periplasmic space, both of which facilitate muropeptide uptake at the cytoplasmic membrane.^76^ In *E. coli*, which is the predominant facultative anaerobe inhabiting the human colon, around 90% of turned-over 1,6-anhydromuropeptides are recycled per generation, and approximately 6–8% shed into the environment in liquid culture conditions.^77^ Peptidoglycan recycling in monoderms is less studied, but is thought to be far less efficient than recycling in diderms, with estimates ranging from 25–50% of turned-over peptidoglycan liberated into the environment per generation.^76,78^ Therefore, if experiments in model organisms are representative of the intestinal niche, one might predict that monoderm gut bacteria naturally shed a greater proportion of peptidoglycan into the gut lumen per generation, compared to diderm bacteria. Nevertheless, peptidoglycan recycling has not been explored among representatives of the vast majority of intestinal commensal bacteria. Furthermore, the relevance of 1,6-anhydromuropeptides in terms of gut peptidoglycan uptake and signaling is not clear. In terms of host signaling, 1,6-anhydromuropeptides are biologically significant, since they include Tracheal Cytotoxin (TCT) a 1,6-anhydromuramyl tetrapeptide responsible for destruction of respiratory tract cilia during *Bordetella pertusis* infection, although the 1,6-anhydroMurNAc moiety itself is not required for toxicity.^79^ 1,6-anhydromuropeptides are recognized by human NOD1, but not by NOD2, possibly because the MurNAc C6 is not available for phosphorylation by *N*-acetylhexosamine kinase (NAGK), reported to be critical for NOD2 stimulation^4,7,80^ (Figure 4). Recently, the cytosolic delivery of *meso*DAP-containing muropeptides, including 1,6-anhydromuropeptides, into skin keratinocytes was linked to the presence of the Slc46a2 transporter, and contributed to psoriatic inflammation.^81^ Slc46a2 expression appears to be low in the gut, but the related MDP transporter Slc46a3 is expressed (Human Protein Atlas proteinatlas.org).^81,82^ In *in vitro* studies, mouse Slc46a3 enhanced responsiveness to both MDP and TCT in the human colorectal cancer cell line HCT-116, but not in human embryonic kidney HEK293T cells.^82,83^ Overall, 1,6-anhydromuropeptides have great potential to act as functionally important muropeptides for signaling between host and gut, that would be particular to diderm bacteria. Specific gut bacteria that inefficiently recycle their peptidoglycan could therefore have a disproportionate influence over signaling via NOD1.^80^

NOD1 signaling can be mediated by the peptide component of peptidoglycan alone, with γ-D-Glu-*meso*DAP as the minimal recognized motif.^5^ *N*-acetylmuramoyl-L-alanine amidases (amidases) are an important class of peptidoglycan hydrolase, associated with bacterial functions such as splitting of the septal peptidoglycan during cell division. Amidases cleave between the L-alanine and the MurNAc lactyl moiety to generate free peptides.^84,85^ Thus, they can generate NOD1 ligands in bacteria with *meso*DAP or L-lanthionine in their peptidoglycan (Figure 4), such as in the case of *Neisseria gonorrhoeae* that releases mainly free tripeptide and muramyl tripeptides into the extracellular environment.^86^ The shedding of immunostimulatory peptidoglycan peptides is not strictly associated with pathogenesis, as even nonpathogenic Neisseria species were found to shed peptidoglycan-derived peptides and muropeptides, the extent of which varied between closely related strains.^87^ Amidase products are recycled via the oligopeptide permease (Opp) system,^88,89^ and so again we find an example where generation of NOD ligands could be regulated by the intrinsic recycling efficiency of the bacterium.

Another potential source of soluble muropeptides comes from the synthesis of peptidoglycan precursors which takes place in the cytoplasm of the bacterium. The first steps involve the addition of uridine diphosphate at the C1 position of MurNAc (forming UDP-MurNAc), followed by the sequential addition of the L- and D-amino acids that form the peptide stem (with the addition of the terminal D-Ala-D-Alanyl dipeptide as a single step to generate UDP-MurNAc-pentapeptide).^90^ Intriguingly, the presence of the UDP carrier group expands the range of peptidoglycan motifs recognized by human NOD2 to include UDP-MDP, and UDP-muramyltripeptides containing *meso*DAP or amidated *meso*DAP, as well as lysine (Figure 4).^5^ Normally the host should not come into contact with UDP-peptidoglycan precursors since they are compartmentalized in the cytoplasm or inner membrane of bacteria and should only be exposed upon lysis. However, an ABC exporter of UDP-peptidoglycan precursors was recently identified in a metagenomic screen of healthy human fecal microbiota, and by ectopic expression of the recombinant transporter in *E. coli*.^91^ Protein homology analysis suggested that the transporter belonged to members of the family *Acutalibacteraceae*. Secretion of UDP-precursors by *E. coli* expressing the ABC transporter was protective in a human intestinal explant infection model and improved inflammatory parameters in a mouse DSS colitis model.^91^ Although it is not yet known whether UDP-peptidoglycan precursors are naturally secreted in the gut by the endogenous host of the ABC transporter system, this system is of interest for its therapeutic potential.

#### Liberation of soluble muropeptides by the host: intestinal lysozyme

Lysozyme is currently the major and only known host peptidoglycan glycosidase secreted into the gut lumen, a process carried out by Paneth cells located in the intestinal crypts.^92–94^ In humans, lysozyme is expressed from a single gene (*lyz*), whilst mice have two lysozyme genes that different in their tissue compartment expression. The Paneth cells secrete a gut specific variant called lysozyme P (LysP) which is expressed from the *lyz1* gene.^94,95^ Lysozyme encoded by a second gene (*lyz2*) produces the variant lysozyme M (LysM), which has a broader expression profile including myeloblasts, immature and mature macrophages, neutrophils, myeloid cells and neurons.^94^ LysP is most likely the major enzymatic activity responsible for solubilizing polymeric peptidoglycan into muropeptides that cross the gut barrier, as sera from intestinal lysozyme knockout mice (*Lyz1*^*-/-*^) possess significantly less NOD1 and NOD2 stimulating activity than sera from wild type mice.^96^ This effect could be rescued by oral administration of NOD1 or NOD2 ligands, indicating that diminished production of soluble muropeptides, and not their transport across the intestinal barrier, was responsible for the loss of activity in *Lyz1* knockout mice. There is also a feedback loop between NOD2 pathway activation and lysozyme trafficking in Paneth cells, suggesting that lysozyme production could be tuned to modulate the abundance of soluble muropeptides generated in the gut, although other microbiota signals also impact on LysP secretion.^97^ Overall, it seems possible that the host could regulate the extent to which soluble muropeptides are generated in the gut lumen via lysozyme-mediated cleavage of the glycan chains, whilst the microbiota is responsible for fine tuning the biological activity of the resulting muropeptides by modulating the peptide stem composition via endogenous or secreted peptidoglycan carboxy- and endopeptidase activities.

Dendritic cells and M-cells can transport whole bacteria across the intestinal barrier through antigen sampling mechanisms.^98,99^ However, these bacteria eventually end up in the phagolysosome compartment. Human monocytes can efficiently take up particulate, polymeric peptidoglycan via actin-dependent phagocytosis, or via a dynamin-dependent, clathrin-independent pathway,^100^ but the insoluble peptidoglycan sacculus must be degraded, through glycosidase activity, to release soluble muropeptides that can be sensed by the host.^101^ Modification of the glycan chains of peptidoglycan can alter their sensitivity to degradation by muramidases such as lysozyme, protecting bacteria from lysis in the gut (Figure 4). Reinforcing this point, peptidoglycan modifications that protect against the action of lysozyme on the sacculus after phagocytosis, limit the release of soluble muropeptides, allowing the bacterium to evade innate immune activation^102,103^ (Figure 4). If lysozyme activity is critical to generate soluble peptidoglycan fragments that enter the host system, then glycan chain modifications that resist lysozyme may represent an innate bacterial mechanism that impacts on systemic peptidoglycan levels. *O-*acetylation of MurNAc (on the C6 hydroxyl group) and de-*N*-acetylation of GlcNAc (i.e. conversion to glucosamine), confer resistance to muramidases.^38,102^ Both types of modification are common in members of major phyla present in the gut microbiota. In monoderm bacteria, *O*-acetylation is performed by the membrane-bound *O*-acetyltransferase (MBOAT) enzyme family (OatA or OatB) and is found mainly within members of the Bacillota (*Bacillales, Lactobacillales, Eubacteriales*), as well as members of the Bacteroidota and Actinomycetota (Eggerthellales, Microccales, Jatrophihabitantales).^104,105^ Diderm bacteria typically use Pat family enzymes for *O*-acetylation of their peptidoglycan, which are found in members of the Pseudomonadota (*Neisseriales*, *Enterobacterales*, *Pseudomonadales*) and Campylobacterota (*Campylobacterales*).^105^ *O*-acetylation of GlcNAc can protected against *N*-acetylglucosaminidase mediated autolysis.^38^ Host enzymes with *N*-acetylglucosaminidase activity, (which cleaves the glycosidic bond between GlcNAc C1 and MurNAc C4) have not been reported in the gut, but have been reported in human spleen and kidney, and in bone-marrow derived macrophages.^106–108^ Therefore, if GlcNAc *O*-acetylation plays any particular role against peptidoglycan hydrolases in the gut, it is likely to provide protection against *N*-acetylglucosaminidase activity of enzymes secreted from other members of the flora, such as competing bacteria or phage lysins, and this remains to be explored.^109^

Substantial glycan chain modifications that confer protection against lysozyme have been reported amongst common gut commensal bacteria. For example; *Bacteroides fragilis*, *B. ovatus*, and *B. thetaiotaomicron* have A1γ-type peptidoglycan with 56–66% MurNAc *O*-acetylation, whilst the A1γ-type peptidoglycan of *Akkermansia muciniphila* displays both GlcNAc de-*N*-acetylation and MurNAc *O*-acetylation, the former representing approximately 87% of glucosamine residues.^55,60^ De-*N*-acetylated muropeptides were also detected directly from peptidoglycan isolated from mouse intestinal microbiota, suggesting that these modifications are relatively common.^61^ Future studies should investigate the prevalence of peptidoglycan modifications throughout the microbiota, their importance for survival in the gut environment, and their impact on systemic peptidoglycan levels in the host.

### Modulation of the soluble peptidoglycome composition by the intestinal microbiota

#### Generation of muramyldipeptide

Secreted DL-endopeptidases have received significant attention as a group of peptidoglycan hydrolyzing enzymes active in the gut. Their significance stems from the fact that they cleave the peptide stem between the second and third amino acid position, generating muramyldipeptide moieties, which are the agonists of NOD2 (Figure 4). As NOD2 activation by gut peptidoglycan is frequently linked to homeostatic, protective and beneficial effects for the host, the generation of muramyldipeptide can be considered one of the most important peptidoglycan-related intestinal functions of the gut microbiota. Metagenomic analysis of predicted secreted peptidoglycan hydrolase sequences from human gut bacteria found that members of the Bacillota encoded the majority (approximately 90%) of intestinal secreted DL-endopeptidases.^110^ Accordingly, beneficial secreted DL-endopeptidases have been characterized from the Bacillota, order Lactobacillale, including several species of *Enterococcus* and *Lactobacillus*. Secreted antigen A (SagA) is an NlpC/p60 domain-containing secreted DL-endopeptidase found in all sequenced strains of *E. faecium*, as well as strains of *E. durans*, *E. hirae* and *E. mundtii*, but not in *E. faecalis* or *E. gallinarum*.^111–113^ Among the lactobacilli, *L. salivarius* is reported to secrete the DL-endopeptidase UC118, which has less than 15% identity to other known DL-endopeptidases,^110^ and several species of lactobacilli are reported to secrete a bifunctional peptidoglycan hydrolase, named LPH, which has both *N*-acetyl-β-D-muramidase and DL-endopeptidase activities.^114^ In principle, this dual activity means that LPH could directly generate soluble muramyldipeptides from peptidoglycan, whereas SagA and UC118 are dependent on the activity of a second enzyme with glycosidase activity to cleave the glycan backbone, a function that could be performed by host lysozyme. A pertinent question is whether secreted DL-endopeptidases principally hydrolyze the endogenous peptidoglycan of the enzyme-producing bacterium, or by hydrolyzing the exogenous peptidoglycan from other species of bacteria occupying the intestinal niche. If the latter case is true, then the level of muramyldipeptide in the gut would depend both on the abundance DL-endopeptidase producing bacteria, and the abundance of bacteria that present the preferred substrate peptidoglycan. *In vitro* studies of SagA activity toward different peptidoglycan types have provided somewhat inconsistent results between native and ectopic expression strains.^110,112,115^ Nevertheless, recent advances such as the generation of *E. faecium* and *Lactobacillus* secreted DL-endopeptidase deletion and active site mutants^112,114,116^ and studies of structural and functional requirements of recombinant enzymes *in vitro* ^112^ are steps that should help to clarify the substrate specificity of these enzymes in the future.

Consistent with their role as enzymes that generate NOD2 agonist, SagA, UC118 and LPH have been shown to protect against inflammation and promote healthy gut barrier function in a variety of disease models, using different expression vectors and animal models. SagA was shown to prime the host immune system and enhance gut barrier function against infection with *Salmonella* Typhimurium in *Caenorhabditis elegans* nematodes and in mice.^111,115^ SagA, UC118 and LPH were found to protect against DSS- and/or TNBS-induced colitis in mice.^110,114^ Jang and coauthors further showed that, not only do enterococci that secrete SagA protect against DSS colitis, but that their absence promotes intestinal dysbiosis and inflammation via a negative feedback loop.^117^ They showed that enterococci are particularly sensitive to killing by the peptidoglycan-binding REG3 lectins, and upon DSS-induced intestinal inflammation, increased REG3 expression led to the loss of DL-endopeptidase secreting *Enterococcus* species from the gut. The loss of MDP-generating bacteria dampened NOD2 stimulation, which in turn caused a decrease of IL-22 mediated epithelial tissue repair, increased inflammation and further promoted harmful overproduction of REG3.^117^ Such inflammation due to loss of NOD2-agonist sensing is reminiscent of inflammation in Crohn’s Disease (CD) context, as polymorphisms linked with loss of NOD2 function are the most important genetic risk factor for CD (although not required for CD).^118,119^ Decreased abundance of Bacillota has been reported in CD cohorts, and Gao *et al*. highlighted that members of the Clostridiales linked to this decrease (*Ruminococcaceae* family and *Roseburia* and *Faecalibacterium* genera) included those predicted to encode DL-endopeptidases. They made the intriguing hypothesis that low DL-endopeptidase abundance in the gut microbiota of some Crohn’s Disease (CD) patients might phenocopy the effect of CD-associated NOD2 polymorphism, due to diminished NOD2 agonist production by the gut microbiota.^110^

Beneficial effects of intestinal DL-endopeptidase activity have also been demonstrated in the contexts of tumor development and cancer therapy. Oral administration of LPH reduced inflammation and tumor development in an azoxymethane/dextran sulfate induced mouse model of colitis-associated colorectal cancer.^114^ Meanwhile, secretion of SagA by intestinal enterococci was demonstrated to have an adjuvant effect on immunotherapy targeting immune checkpoint inhibitors, adding to growing evidence that *Enterococcus* populations in the gut are important mediators of the host response to cancer therapy.^120–124^ Taken together, these studies indicate that future research needs to carefully assess the correlation between DL-endopeptidase expression by gut microbiota, and resilience against pathology or responsiveness to therapies in cancer context, and in the contexts of IBD and chronic systemic inflammatory diseases. We can also speculate that within the gut, the balance between the generation of soluble peptidoglycan fragments that signal via NOD1 or NOD2, is tipped in favor of NOD2 stimulation thanks to the presence of secreted DL-endopeptidases. In this regard, it is worth noting that muramyl dipeptides escape cleavage by the serum amidase PGLYRP-2, and thus the host system seems permissive to signaling via DL-endopeptidase products.^125^ It may be the case that co-evolution of microbiota and host has led the healthy gut to anticipate the presence of DL-endopeptidase secreting bacteria and, as a consequence, their absence has repercussions on host physiological responses and health.

#### Generation of muramyltripeptides

The major characterized ligands for human NOD1 require *meso*DAP (or *meso*lanthionine) as the diamino acid in the C-terminal position of the peptide, which necessitates the involvement of LD-carboxypeptidase activity that cleaves between the third and fourth amino acids on the peptide stem. NOD2 agonists also include muramyltripeptides (e.g. MTriLys, MTriOrn)^5^ (Figure 4). Thus, an obvious question is whether LD-carboxypeptidases are secreted by gut bacteria to modulate host homeostasis or disease effects by generating NOD1 and NOD2 ligands. There is also a potentially significant role for DD-endopeptidase enzymes that hydrolyze the crosslink between peptide stems or remove bridge peptides from the muramyl tripeptide stem. Metagenomic analysis has predicted LD-carboxypeptidase genes in members of all gut bacteria phyla except the Actinomycetota (Actinobacteria) where detection was rare.^110^ High copy numbers of DD-endopeptidase were predicted in gut Pseudomonadota (Proteobacteria), lower copy numbers in Bacteroidota (Bacteroides), and a sparse presence among the Bacillota (Firmicutes).^110^ Whether any of these enzymes are secreted to act on exogenous peptidoglycan and significantly influence the abundance of gut muramyltripeptides remains to be determined. However, an important role for muramyltripeptide generation through autologous peptidoglycan hydrolysis in the gut has been demonstrated, since lysine-containing muramyltripeptide of *Lactobacillus salivarius* peptidoglycan was shown to protect against TNBS-induced colitis in mice, via NOD2-mediated immunosuppression.^126^ Microbiota peptidoglycan was also shown to mediate homeostasis of human pancreatic beta cells via NOD1.^96^ Therefore, future studies into peptidoglycan LD-carboxypeptidase enzymes in the gut should shed additional light on the relationship between microbiota peptidoglycan and NOD1/NOD2 mediated homeostasis. An added complication to consider, is that mouse NOD1 is activated by muramyl tetrapeptide containing *meso*DAP, and this is why transgenic mice expressing human NOD1 have been used to probe microbiota peptidoglycan hydrolase activities that are relevant to human health in murine models.^127,128^

### Selection at the gut barrier

In the steady state, soluble muropeptides must be trafficked across the gut epithelial barrier to reach the host system.^61,129–131^ Precisely how peptidoglycan crosses the gut epithelial barrier under homeostatic conditions, and whether selectivity toward specific muropeptides takes place at the epithelial barrier is poorly understood. One possible uptake mechanism is the passive diffusion of muropeptides across the gut barrier via paracellular transport. In the absence of epithelial damage, paracellular transport occurs through two possible pathways. The first is the pore pathway, mediated by pore-forming claudin proteins within the epithelial tight junctions. In the gut, the pore pathway permits passive translocation of small cations less than 0.6 nm in diameter and is unlikely to accommodate even the smallest muropeptides (reviewed in^132^). A second pathway, the leak pathway, involves active remodeling of the epithelial tight junctions and accommodates the paracytosis of molecules with a diameter of up to 12.5 nm, with no charge selectivity. This mechanism is regulated by Na^+^-nutrient cotransport, which activates long myosin light chain kinase splice variant 1 (MLCK1), triggering remodeling of the epithelial tight junction by endocytosis of occludin. The increased paracellular permeability allows greater water absorption, with simultaneous uptake of epithelium-adjacent solutes via solvent drag.^132^ The leak pathway thus represents a potential nonselective pathway for trafficking muropeptides across the epithelial barrier. In germ free mice, gut epithelial tight junction claudins and occludin are more highly expressed than in conventional microbiota mice, meaning that paracellular permeability is lower in the absence of microbiota.^133^ Our recent data show that peptidoglycan absorption across the gut barrier is suppressed in germ free mice, which would fit a leak pathway model.^61^ Another possibility is active transcytosis by gut epithelial cells, with uptake mediated by membrane transporters (Figure 4). Members of the SLC15A, SLC46A and pannexin families, are candidates to bring gut muropeptides across the luminal surface of the intestinal epithelial barrier.^81,83,134–140^ If membrane transporters represent the major muropeptide uptake system, then their selectivity toward specific muropeptide substrates would essentially determine the diversity of the “systemic peptidoglycome” at homeostasis. For example, SLC15A-family oligopeptide transporter PepT1 transports di- and tripeptides, but not amino acids or longer peptides. Consistent with this, muramyl di- and tripeptides have been characterized as substrates of PepT1. If PepT1 is the major muropeptide transporter active in the gut, then it would essentially restrict the diversity of muropeptides reaching the host system to muramyl di- and tripeptides, that are recognized by the NOD1 and NOD2 receptors. The substrate range of the SCL46A family transporters may be a little broader, as it seems to also accommodate muramyl tetrapeptides.^81^ Of course, nonselective and selective uptake systems could be active simultaneously. For example, the leak pathway could permit a basal level of nonspecific paracytosis, with enrichment of specific muropeptides via membrane transporter mediated transcytosis. Careful studies comparing the diversity of soluble muropeptides in the intestinal lumen, versus the diversity of muropeptides in serum, would shed light on the relative contribution of paracellular transport versus membrane transporter mediated transcytosis as mechanisms of peptidoglycan transfer across the intestinal barrier. Furthermore, whether epithelial damage results in the unrestricted diffusion of MAMPs into the host system, including muropeptides that would not normally be abundant systemically, remains to be carefully explored and could be a factor linking intestinal dysbiosis and gut peptidoglycan-mediated effects of systemic chronic inflammatory disease.

### Modulation of the systemic peptidoglycan composition

Little is known about the metabolism of peptidoglycan within the host system, mainly due to the difficulty of identifying and directly studying muropeptides from host biomaterials. The major enzyme known to modulate muropeptide structures systemically is peptidoglycan recognition protein 2 (PGLYRP-2), an *N*-acetylmuramoyl-L-alanine amidase secreted abundantly from the liver into the blood stream.^141^ For this reason, it is referred to as the “serum amidase”. Amidases cleave the bond between MurNAc and L-alanine in muropeptides with the minimal recognition motif being muramyl-tripeptide, and it should therefore be noted that MDP escapes PGLYRP-2 amidase activity.^125,142^ Although MurNAc is dispensable for activation of NOD1 by muropeptide ligands, the presence of the MurNAc moiety is essential to the bioactivity of NOD2 ligands.^5^ Along with secretion into the blood, PGLYRP-2 expression is reported in CD3+ CD11c+ intraepithelial T lymphocytes in the gut, and amongst populations of splenic T cells. Expression in different tissues may be broader but context dependent, as PGLYRP-2 was found to be expressed in keratinocytes only upon exposure to bacteria or appropriate immune signals.^143,144^ PGLYRP-2 is proposed to cleave and inactivate systemic NOD2-agonistic muramyltripeptides derived from gut bacteria. In other words, it is poised to specifically dampen the activity of muropeptides containing the diamino acids L-lysine or L-ornithine. A further hypothesis is that PGLYRP-2 restricts the sphere of influence of these NOD2-agonistic muramyltripeptides locally to the mucosal tissues at the interface with the microbiota. The secretion of amidases in the gut, and the potential consequences for the host, has not received much attention. Metagenomic analysis indicated that amidases are widely dispersed among the members of the gut microbiota.^110^ If members of the gut microbiota secrete amidases, this activity could dampen NOD2-signaling at the level of in intestine. Furthermore, since the substrates may not be restricted to muramyltripeptides and larger peptidoglycan fragments, but could potentially include MDP, then such an activity would act to dampen NOD2 signaling locally in the gut and systemically. As such, amidase activities of gut microbiota deserve further study.

There are few other clues regarding the metabolism or catabolism of peptidoglycan fragments within the host system. A study by Valinger *et al*. found that after oral administration of ^14^C-labeled peptidoglycan to mice, radioactivity was detected in exhaled CO^2^ 48 h later, suggesting complete breakdown of the peptidoglycan molecule *in vivo*, and potential direction into host metabolic pathways.^145^ Although the bulk of orally administered peptidoglycan is cleared within 7–8 h of gavage, radiolabeled peptidoglycan can be detected in the host circulation and intestinal tract at basal levels for up to 72 h post gavage.^129^ Thus, it is also likely that a proportion of orally administered peptidoglycan is taken up by the gut microbiota itself and is potentially recycled or processed into non-peptidoglycan substrates. Therefore, care must be taken when using labeling approaches to ensure that processing of the peptidoglycan by the host can be separated from its processing by the microbiota into new non-peptidoglycan metabolites that could also be absorbed by the host.

### The tip of the iceberg: impacts on the host reveal physiological roles for gut microbiota peptidoglycan

#### PRR mutations highlight potential roles of peptidoglycan in the host

Frequently, physiological roles of peptidoglycan are revealed when steady-state sensing of peptidoglycan breaks down and manifests as disease. The classical example is Crohn’s Disease, where mutations in the leucine-rich repeat domain of *CARD15*(*NOD2*), involved in ligand binding, represent the greatest genetic risk factor for development of the disease (although *CARD15* mutation is neither necessary nor sufficient for the occurrence of Crohn’s Disease).^118,119,146^ With the identification of NOD2 mutations (*CARD15* gene) as a major risk factor, came the implication that the steady-state sensing of gut peptidoglycan by NOD2 is part of healthy gut barrier homeostasis. Subsequent studies showed that NOD2 stimulation can suppress inflammation and promote gut health, often demonstrated using the 2,4,6-trinitrobenzene sulfonic acid (TNBS) model of experimental colitis, which mimics the protective requirement of functional NOD2 associated with Crohn’s Disease.^110,126,147–149^ Since this time, NOD2 activation has been associated with promoting epithelial repair, antimicrobial peptide secretion, immune tissue development, immune priming, and modulation of NF-κB activation by other PRRs,^150^ all of which promote a healthy gut barrier, and dysbiosis of which would contribute to harmful inflammation.

Subsequently, specific alleles of *CARD15* have been identified as genetic risk markers for a diverse range of diseases. The strongest links to disease involve chronic autoinflammatory disorders. Specific mutations in the *CARD15* gene result in the monogenic disease Blau syndrome and its sporadic form Early Onset Sarcoidosis, characterized by dermatitis, arthritis and uveitis.^151^ Blau syndrome is thought to result from a gain-of-function mutation of NOD2, as HEK293 cells expressing *CARD15* Blau risk alleles demonstrated increased basal NF-κB activation in the absence of MDP stimulation.^152^ Blau-associated NOD2 variants are also less responsiveness to MDP, demonstrated in transgenic mice expressing Blau *CARD15* alleles, and using primary macrophages from patients.^153^ Similarly, Yao syndrome is an autoimmune disease manifesting in bouts of fever, dermatitis, arthritis and gastrointestinal inflammation, associated with NOD2 variants.^154,155^

Although risk alleles in *CARD4*(*NOD1*) and *CARD15* receptors have not been reported in rheumatoid arthritis context, there is a strong association between elevated levels of systemic peptidoglycan and arthritis. Higher levels of peptidoglycan have been detected in the blood or synovial tissues of rheumatoid arthritis and systemic lupus erythematosus patients relative to controls,^130,156,157^ and systemic administration of a neutralizing antibody targeting MDP was protective in a murine collagen-induced arthritis model.^130^ Further evidence that systemic peptidoglycan can mediate arthritis inflammation comes from the observation that injection of MDP systemically or into the joints of BALB/c mice is sufficient to induce acute transient arthritis.^158,159^ NOD2 and PGLYRP-2 are required for peptidoglycan-induced arthritis, whilst PGLYRP-1 is protective.^160^ In the case of *Borrelia burgdorferi* acute infectious arthritis, the unusual peptidoglycan (A3β) of the bacterium appears to resist clearance and persist in tissues to induce arthritis in the absence of live bacteria.^64^ This study raises the question as to whether the pharmacokinetics of specific muropeptide structures entering the host system from the gut could be a factor in chronic inflammatory diseases such as arthritis. Together these studies would suggest that, in the healthy host, gut peptidoglycan uptake and elimination mechanisms must be tightly regulated to maintain basal levels of systemic peptidoglycan that facilitate steady-state functions without triggering pathological inflammation. Precisely how gut peptidoglycan is selected, absorbed and cleared from the host, and how these processes are regulated is perhaps the least understood aspect of the peptidoglycome iceberg.

Neurodegenerative and psychiatric disorders have also been linked to peptidoglycan-sensing risk alleles. Variants of PGLYRP-3 and PGYLRP-4 have been associated with risk of Parkinson’s Disease,^161^ as have NOD2 alleles,^162,163^ although this has been disputed.^164^ However, studies in a neurotoxin-induced model of Parkinson’s Disease found that stimulation of NOD2 in microglia led to the degeneration of dopaminergic neurons.^165^ Conversely, in the case of an Alzheimer’s Disease mouse model, treatment with MDP induced therapeutic immunomodulation of monocyte populations and delayed onset of memory impairment.^166^ Risk alleles of *CARD15* have also been associated with mental illness including bipolar disorder^167^ and schizophrenia.^168^ It is interesting to note that sleep disorders are associated with mental illness, and some the earliest studies that identified physiological effects of peptidoglycan were related to sleep alterations.^169^

Whereas *PGLYRP3* and *PGLYRP4* risk alleles are linked to disease, *PGLYRP2* knock-out mice exhibit age and sex dependent behavioral alterations, centered around anxiety behaviors and motor function, associated with altered neurochemistry.^170^ However, whether any of the observed behavioral changes were dependent on altered sensing or hydrolysis of peptidoglycan remains to be established. An interesting study by Humann *et al*. raises the possibility that bacterial cell wall fragments including peptidoglycan could affect the architecture of the brain. Mimicking the pathological context of streptococcal meningitis, Humann *et al*. found that bacterial cell wall (peptidoglycan retaining phosphorylcholine and teichoic acids) was capable of crossing the mouse placental barrier and reaching the fetal brain through a mechanism that involved binding to platelet activating factor (PAFr).^171^ This resulted in abnormal, TLR2-dependent proliferation of neurons, leading to impaired memory and cognitive function in pups. Although the observed effects were likely not specific to peptidoglycan, which acted rather as a polymeric carrier scaffold, both NOD1 and muropeptide transporter PepT2 appear to be expressed in the placenta.^172^ Thus, we can ask whether muropeptides from the mother’s gut microbiota could translocate the placental barrier and influence development of the fetal brain or other physiological aspects, and what role microbiota peptidoglycan might play in development during early life when expression of peptidoglycan PRRs in the brain appears to be particularly malleable.^173^

#### Peptidoglycan homeostasis – a matter of life and death

In drosophila, muropeptides shed by gut bacteria translocate into the hemolymph (the insect analogue of blood), from where they can reach organs and interact with the immune system, and are recognized by peptidoglycan recognition proteins to trigger Toll- or Imd-mediated inflammation. Homeostasis is maintained by nephrocytes that remove excess peptidoglycan from the hemolymph. By studying nephrocyte deficient flies, Troha *et al*. revealed a trade-off between Imd-dependent resistance to infection and the lifespan of flies.^174^ Failure to clear elevated systemic peptidoglycan led to chronic over-priming of the toll-inflammatory pathway, that rendered the flies highly resistant to infection. However, the cost to flies was a reduced lifespan in the absence of infection. Onuma *et al*. also observed a trade-off between lifespan and resistance to infection via Imd pathway activation, that was differentially regulated by peptidoglycan from diderm (potent PGRP-LC-mediated Imd activation) versus monoderm (mild and spatially restricted PGRP-LE-mediated Imd activation) bacteria, despite all peptidoglycan types containing *meso*DAP.^175^ The likely explanation is the prominence of 1,6-anhydromuropeptides derived from the peptidoglycan of diderm bacteria, as peptidoglycan receptors PGRP-LC and PGRP-LE are capable of sensing TCT (specifically, GlcNAc-1,6-anhydromuramyl tetrapeptide).^176,177^ Recently, Fioriti *et al* demonstrated that TCT induced chronic Imd-activation in the brain, that led to early death of flies due to progressive neurological impairment that was exacerbated by infection in older flies. The authors showed that TCT translocated from the gut to the brain, potentially activating the Imd pathway directly in glial cells and neurons to induce immune defenses.^178^ Intriguingly, TCT could not be detected in the brain when injected directly into the hemolymph, reminiscent of our observation that radioactively labeled peptidoglycan was detected in the brain of mice when administered by gavage, but not intravenous injection, which suggests that a gut-dependent mechanism may facilitate microbiota peptidoglycan trafficking to the brain.^61,178^ Together, these studies show that in drosophila, gut peptidoglycan sensing has evolved as part of a delicate balance between immune defenses that protect against infection, and inflammatory stress that is harmful to the host.

Whilst gut peptidoglycan homeostasis is a matter of life and death in flies, in more complex vertebrate organisms, steady-state gut peptidoglycan signaling can influence the lifespan of specific cellular compartments through NOD1 and NOD2. Hergott *et al*. showed that steady-state NOD1 stimulation regulated the rate of apoptosis and turnover of peripheral circulating neutrophils and Ly6C+ inflammatory monocytes, but not other cell populations such as lymphocytes, fibroblasts or endothelial cells.^179^ In this case, peripheral phagocytes did not sense systemic peptidoglycan fragments directly. Instead, intestinal lymphocytes secreted IL-17A as a survival signal in response to NOD1 stimulation. Microbiota peptidoglycan is also a signal for cell proliferation and tissue development within the intestinal tract, as NOD1 stimulation by microbiota peptidoglycan was the major developmental trigger for formation of B-cell rich lymphoid follicles.^180^ Meanwhile, also in the gut niche, microbiota peptidoglycan is a survival signal for Lgr5+ stems cells that express NOD2 in the intestinal crypts, stimulating cell proliferation, tissue repair and resistance to oxidative stress.^181^ Direct peptidoglycan effects on immune cells in the steady state are not restricted to peptidoglycan interactions with the gut epithelia. Gut peptidoglycan was demonstrated to translocate to the bone marrow in mice, where activation of NOD1 primed neutrophils against bacterial infection. Intriguingly, NOD1-primed neutrophils became more bactericidal toward monoderm bacteria with peptidoglycan that lacked NOD1 ligands.^129^

#### Gut microbiota peptidoglycan as a diet and energy homeostasis regulatory signal

Recent studies have connected gut peptidoglycan to host behaviors related to food consumption, with behavioral alterations observed across invertebrate and vertebrate models. *Caenorhabditis elegans* is a soil nematode that feeds on bacteria and fungi. Under sterile laboratory conditions, the consumption of peptidoglycan acts as a feeding signal that influences attractiveness of food sources, and stimulates digestive processes, allowing the nematodes to thrive on previously inedible bacteria.^182,183^ None of these effects seem to involve traditional invertebrate peptidoglycan PRRs. However, they depended on the structure of the consumed peptidoglycan, as the effects were lost upon treatment of peptidoglycan with amidase or *N*-acetyl-D-glucosaminidase, when peptidoglycan was purified from mutants affected in their peptidoglycan metabolism, or when *E. faecalis* (A3α) peptidoglycan was used as a feeding supplement (conversely *E. coli* and *B. subtilis* (A1γ) peptidoglycan and lysozyme digested peptidoglycan were effective). Together these data provide tantalizing clues on the core structural motifs that may be required for peptidoglycan-mediated digestive signaling in nematodes.^182,183^ In mice, rather than stimulating feeding, peptidoglycan acts as a feedback signal to suppress appetite. In older female mice, orally administered MDP was found to translocate to the brain, and directly stimulate NOD2 in inhibitory (Vgat^+^) neurons of the mouse hypothalamus. The resulting behavioral alterations included reduced appetite, reduced body temperature and, consequently, increased nest-building activity.^131^ Why might peptidoglycan be used by the host as an appetite feedback signal? When a host consumes a meal, they are also providing nutrients for the growth of the gut microbiota. As such, the overall abundance and composition of the microbiota undergoes diurnal oscillations that are determined by the feeding patterns of the host.^184^ We speculate that oscillations in microbiota growth and clearance are also likely to provide circadian oscillations in peptidoglycan fragment generation by the gut microbiota, due to changes in the metabolic activity of the gut flora in the presence and absence of nutrients. All four mammalian PGLYRP peptidoglycan sensors, as well as NOD1 and NOD2, have been detected as PRRs with specific spatial and temporal expression profiles in the mouse brain, and peptidoglycan has been detected in brain tissue in numerous studies,^61,131,173,185^ indicating that there is ample opportunity for gut peptidoglycan signaling within different brain tissue compartments, with unknown consequences for the host. This raises the question of how gut peptidoglycan reaches and crosses the blood brain barrier. To this end, PepT1 has been highlighted as a muropeptide transporter that could potentially perform this function in the brain, and MDP transporter SLC46a3 is reported to be well expressed (Human Protein Atlas.org).^81,173^

Circulating peptidoglycan from the gut microbiota plays a direct role in energy metabolism. In a malnourished postnatal mouse model, NOD2 stimulation in intestinal epithelial cells lead to improved body size, bone growth, and levels of circulating insulin and insulin-like growth factor-1.^186,187^ Both NOD1 and NOD2 were previously suggested to regulate bone density^188^ and have an important role in insulin homeostasis. Systemic peptidoglycan regulates homeostasis of insulin trafficking via NOD1 signaling in pancreatic islet beta cells, which triggers the recruitment of Rab1a to direct the trafficking of insulin vesicles.^96^ High fat diet has been demonstrated to induce increased systemic circulation of NOD1 and NOD2 ligands, and this can promote chronic overstimulation of NOD1, contributing to the development of insulin resistance and inflammation, whilst NOD2 stimulation can suppress these effects via a pathway mediated by the effector molecule IRF4.^189,190^ In the malnourished mouse model, specific strains of *Lactobacillus plantarum* alleviated the effects of malnourishment, and the peptidoglycan of protective *L. plantarum* strains was sufficient to induce protection. Intriguingly, only subtle differences in the extent of *meso*DAP amidation and *O*-acetylation were observed in the peptidoglycan of protective versus non-protective *L. plantarum* strains.^187^ Since lysozyme (but not mutanolysin) is inhibited by *O*-acetylation, this study raises the possibility that subtle differences in *O*-acetylation might lead to a physiologically significant impact on the liberation of NOD2 ligands by lysozyme, an aspect that will require further study.^96^

## Conclusion

Peptidoglycan has an enormous influence on human health, and at its foundation is the intestinal peptidoglycome. This review highlights the ongoing need for fundamental studies toward a mechanistic understanding of the interactions that shape the intestinal peptidoglycome, and the pathways through which peptidoglycan interacts with the host immune and other physiological systems. If we were to highlight one question as being the major unknown, and research priority within the hidden base of the peptidoglycome iceberg, it is “precisely how do peptidoglycan fragments cross the gut barrier?” Why is this the most critical question? The gut microbiota may release hundreds of different peptidoglycan fragments, or we may find that very few are released in sufficient proportion to be of physiological relevance to the host. But if peptidoglycan fragments cannot reach host peptidoglycan sensing receptors, then their zone of influence is restricted to the gut lumen. The degree of selectivity inherent to peptidoglycan uptake mechanisms at the gut epithelial barrier is likely to be an important border-check through which the host permits or prohibits specific muropeptides to enter the host system in the steady-state. Of course, disruption of the epithelial barrier integrity would override regulated uptake of peptidoglycan fragments, leading to dysregulated responses to peptidoglycan with harmful consequences for the host. Therapeutic interventions would benefit immensely from understanding which peptidoglycan fragments are permitted, and which are excluded to cross the gut barrier in a healthy host. Our recent work indicated that peptidoglycan uptake is modulated by the composition of the microbiota,^61^ hinting at plasticity in the uptake systems that can be probed to uncover both the precise uptake pathways and their specific regulatory mechanisms. One of the major limitations for study of peptidoglycan uptake systems *in vivo*, is the lack of tools dedicated to identification and quantification of peptidoglycan fragments from host biomaterials. Currently, this technological shortfall appears to be the subject of intense development.^60,73,191,192^ Thus, it seems that the field is primed for major advances that will provide new insights into the dynamic relationship between host and gut microbiota in health and disease, and how the gut peptidoglycome can be manipulated toward therapeutic needs.
